# Enhancing the Functional and Emulsifying Properties of Potato Protein via Enzymatic Hydrolysis with Papain and Bromelain for Gluten-Free Cake Emulsifiers

**DOI:** 10.3390/foods14060978

**Published:** 2025-03-13

**Authors:** Wen-Chieh Sung, Chui-Xuan Tan, Pei-Hsuan Lai, Shang-Ta Wang, Tai-Ying Chiou, Wei-Ju Lee

**Affiliations:** 1 Department of Food Science, National Taiwan Ocean University, Keelung 20224, Taiwan; sungwill@mail.ntou.edu.tw (W.-C.S.); 11132041@mail.ntou.edu.tw (P.-H.L.); wst@mail.ntou.edu.tw (S.-T.W.); 2 Center of Excellence for the Oceans, National Taiwan Ocean University, Keelung 20224, Taiwan; 3 School of Food Safety, Taipei Medical University, Taipei 11031, Taiwan; ma47111001@tmu.edu.tw; 4 Institute of Food Safety and Risk Management, National Taiwan Ocean University, Keelung 20224, Taiwan; 5 School of Regional Innovation and Social Design Engineering, Kitami Institute of Technology, Hokkaido 090-8507, Japan; tkyuu@mail.kitami-it.ac.jp

**Keywords:** potato protein, enzymatic hydrolysis, papain, bromelain, emulsifying property

## Abstract

In recent years, plant-derived food proteins have gained increasing attention due to their economic, ecological, and health benefits. This study aimed to enhance the functional properties of potato protein isolate (PPI) through enzymatic hydrolysis with papain and bromelain, evaluating the physicochemical and emulsifying characteristics of the resulting potato protein hydrolysates (PPHs) for their potential use as plant-based emulsifiers. PPHs were prepared at various hydrolysis times (0.25–2 h), resulting in reduced molecular weights and improved solubility under acidic conditions (pH 4–6). PPHs exhibited higher ABTS radical-scavenging activity than PPI. The foaming stability (FS) of bromelain-treated PPI was maintained, whereas papain-treated PPI showed decreased FS with increased hydrolysis. Bromelain-treated PPHs demonstrated a superior emulsifying activity index (EAI: 306 m^2^/g), polydispersity index (PDI), higher surface potential, and higher viscosity compared to papain-treated PPHs, particularly after 15 min of hydrolysis. Incorporating PPHs into gluten-free chiffon rice cake batter reduced the batter density, increased the specific volume, and improved the cake’s textural properties, including springiness, cohesiveness, and resilience. These findings suggest that bromelain-treated PPHs are promising plant-based emulsifiers with applications in food systems requiring enhanced stability and functionality.

## 1. Introduction

The plant-based food market is experiencing rapid growth, driven by increasing demand for plant-derived alternatives to animal-based products such as eggs, meat, milk, and their analogs [1]. As a result, scientists are exploring novel plant-based ingredients to meet these needs. The potato, the world’s fourth most important crop after wheat, rice, and corn, with an annual production exceeding 300 million tons, holds significant potential in this context [2]. The potato starch industry generates a substantial amount of potato juice as a byproduct, which contains approximately 25 g of protein/kg [3]. Potato protein is garnering increasing attention due to its superior nutritional profile compared to other plant proteins, particularly its higher lysine content and better solubility and digestibility compared to wheat protein [4,5,6]. Moreover, potato protein has demonstrated beneficial health effects, including antimicrobial, antioxidant, blood pressure regulation, and cholesterol-lowering properties [7,8,9,10]. Additionally, potato protein is non-allergenic, making it a promising ingredient for the food industry [11].

Potato protein can be classified into three main categories: (1) patatin, which constitutes 40% of the total protein, has a molecular weight of 40–45 kDa, exists primarily as an 80 kDa dimer, and exhibits different isoelectric points (pI 4.5–5.2) and glycosylation patterns; (2) protease inhibitors, which make up approximately 50% of the total protein and have a molecular weight of around 21 kDa; and (3) high-molecular-weight proteins such as lectins, polyphenol oxidases, protein kinases, phosphatases, and enzymes involved in starch biosynthesis, which account for about 10% of the total protein. The compositions of these proteins may vary depending on the potato variety and growing conditions [4,5,12,13,14]. Patatin, which exists as four isoforms (A–D), possesses enzymatic activities capable of digesting fats [5,15]. It also contains several amino acids with free-radical-scavenging properties that contribute to its antioxidant activity [16]. Furthermore, patatin has demonstrated excellent foaming and emulsifying properties [17,18,19]. Despite these promising attributes, the functional properties of plant proteins, including potato protein, are often inferior to those of animal-derived proteins due to denaturation during protein extraction. Therefore, enhancing the functional properties of plant-based proteins through modifications is crucial for their incorporation into various food products [20].

Enzymatic hydrolysis is recognized as an effective method to improve the functional characteristics of proteins while maintaining their nutritional value [21]. The process is influenced by factors such as enzyme types, substrate concentrations, enzyme concentrations, temperature, pH, and reaction times [22,23]. Controlled enzymatic hydrolysis can enhance protein solubility, oil and water retention, emulsifying properties, foaming abilities, gelation, and sensory characteristics. Additionally, this process can improve the shelf life and purity of the protein, while reducing environmental impacts [24,25,26]. Limited enzymatic hydrolysis, characterized by lower enzyme concentrations and mild reaction conditions, is preferred over excessive hydrolysis, which can lead to the loss of functional properties [27,28,29]. Proteases commonly used in protein hydrolysis include pepsin, alkaline protease, papain, trypsin, and bacterial and fungal proteases [30]. Recent studies showed that hydrolyzed proteins and peptides obtained through enzymatic hydrolysis possess enhanced functional properties and health benefits, such as antioxidant and antimicrobial activities [31,32].

Alcalase-hydrolyzed potato protein, which consists mostly of subtilisin A, was reported to possess emulsifying and antioxidant properties, and its incorporation in sausages at 2.5% was found to reduce the fracture force and cooking losses of frankfurters [33]. Potato protein hydrolysates obtained via alcalase hydrolysis had significantly improved emulsion stability and activity [34]. Additionally, potato protein isolates (PPIs) hydrolyzed with enzymes like Flavourzyme (*Aspergillus oryzae*), Neutrase (*Bacillus amyloliquefaciens*), and bromelain exhibited smaller particle sizes, higher solubilities, and antioxidant potential, making them suitable for medical applications [35]. Papain and bromelain, plant-derived endopeptidases, are widely used in the food industry, particularly as meat tenderizers [36,37], and in pharmaceutical preparations [36,38]. Bromelain refers to a complex mixture of proteolytic enzymes primarily extracted from pineapple. Similarly, papain is a broad term denoting a group of proteolytic enzymes. However, few studies have focused on the hydrolysis of potato protein by papain and bromelain.

Several studies have investigated the potential of plant-derived proteins as emulsifiers in various food products, including cakes, ice cream, and salad dressings [39]. However, the production of leavened non-gluten baked goods requires the incorporation of hydrocolloids, emulsifiers, and proteins to address the technological limitations inherent in rice-flour-based formulations [40]. In light of this, the objectives of the present study were to partially hydrolyze PPIs using papain and bromelain and assess the physicochemical and emulsifying properties of the resulting hydrolysates. Additionally, this study aimed to compare the performances of the modified potato proteins to those of a traditional emulsifier in gluten-free chiffon rice cake formulations. Ultimately, the broader goal was to enhance the functional properties of enzyme-modified potato proteins, paving the way for their potential use in diverse food applications.

## 2. Materials and Methods

### 2.1. Raw Materials and Chemicals

PPI (Solanic^®^ 200) was obtained from Avebe (Veendam, The Netherlands). Bromelain was purchased from Acros Organics (Newark, NJ, USA). Wet-milled Japonic rice flour was sourced from Ping-Tung Foods (Pingtung, Taiwan). Sorbitan fatty acid ester (RIKEMAL SV-690W) was provided by Rikevita Asia (Taipei, Taiwan). Other ingredients, including eggs, sucrose, whole milk, soybean oil, lemon juice, and baking powder, were purchased locally (Taipei, Taiwan). Reagents such as N,N,N′,N′-tetramethylethylenediamine (TEMED), 2-mercaptoethanol, methanol, papain, Folin–Ciocalteu reagent, ascorbic acid, sodium hydroxide, copper sulfate, sodium carbonate, potassium sodium tartrate, acetone, 2,2-diphenyl-1-picrylhydrazyl (DPPH), 2,2′-azino-bis-3-ethylbenzothiazoline-6-sulfonic acid (ABTS), trichloroacetic acid, acetic acid, potassium ferricyanide, and hydrochloric acid were purchased from Sigma-Aldrich (St. Louis, MO, USA), PanReac Applichem (Gatersleben, Germany), J.T. Baker (Bridgewater, NJ, USA), Alfa Aesar (Ward Hill, MA, USA), and Merck (Whitehouse Station, NJ, USA). All reagents were of an analytical grade.

### 2.2. Potato Protein Hydrolysate (PPH) Preparation and Characterization

#### 2.2.1. PPH Preparation

The PPH was prepared by referencing and modifying the methods of Akbari et al. and Chang et al. [34,35]. PPI was hydrolyzed using papain or bromelain. A potato protein dispersion of 100 mg/mL was prepared by dissolving 10 g of PPI in 100 mL of distilled water and heating to 95 °C for 1 h, followed by cooling to room temperature. Enzymes were added at a 1:100 (*w*/*w*) ratio, with papain (≥30,000 USP-U/mg) being incubated at 65 °C and pH 6 for 0.5~4 h, and with bromelain (1200 GDU/g) being incubated at 50 °C and pH 7 for 0.5~4 h. The pH was adjusted using 0.5 M HCl or 0.5 M NaOH. Reactions were terminated by heating to 95 °C for 15 min. The hydrolysates were centrifuged at 11,000× *g* for 20 min at 4 °C, filtered through No. 1 ADVANTEC^®^ filter paper (Toyo Roshi Kaisha, Tokyo, Japan), and freeze-dried for 48 h.

#### 2.2.2. Yield of PPH

The yield was calculated as follows: (W_PPH_/W_PPI_) × 100, where W_PPH_ and W_PPI_ respectively are the freeze-dried weights of the PPH and the initial PPI.

#### 2.2.3. Degree of Hydrolysis

The degree of hydrolysis was determined according to the method of Akbari et al. [34]. The filtered sample from the previous step was mixed thoroughly with 20% trichloroacetic acid (TCA) solution at a 1:1 ratio. The mixture was then centrifuged at 6700× *g* for 20 min at 10 °C. The soluble protein content in 10% TCA solution was measured following the method of Lowry et al. [41], with bovine serum albumin as the standard. The degree of hydrolysis was calculated using the formula:

$$\mathrm{Degree}\;\mathrm{of}\;\mathrm{hydrolysis}=\frac{\mathrm{Soluble protein in}\;10\%\mathrm{TCA}\;(mg/mL)}{Total\;protein\;(mg/mL)}\times 100$$

#### 2.2.4. Molecular Weight

The molecular weights of the PPI and PPHs were analyzed by sodium dodecyl sulfate polyacrylamide gel electrophoresis (SDS-PAGE) using a vertical electrophoresis system (BIO-RAD Mini PROTEAN^®^ Tetra Cell, Hercules, CA, USA). The samples were prepared by being mixed with Laemmli buffer and heated to 100 °C for 5 min. Electrophoresis was run at 80 V for 30 min, then at 120 V for 120 min. The gel was stained with Coomassie brilliant blue R250 and destained with acetic acid.

#### 2.2.5. Solubility

Solubility was assessed by adjusting the pH of 1 mg/mL of the PPI and PPH solutions from pH 3 to 8 with 0.1 N NaOH or HCl. After 30 min of shaking and centrifugation at 10,000× *g* for 30 min, the soluble protein content was measured using the Lowry method. Solubility was calculated as the ratio of the soluble protein content to the total protein content.

#### 2.2.6. Antioxidant Activity

The ABTS radical-scavenging activity was measured as described by Re et al. [42]. The reaction mixture (40 μL of ABTS radicals + 160 μL of sample at a concentration of 2 mg/mL, diluted with water) was incubated in the dark for 5 min at 30 °C, and the absorbance at 734 nm was recorded. The DPPH-scavenging activity was determined by mixing 1 mL of a 10 mg/mL sample with 1 mL of a 0.1 mM DPPH solution. The absorbance at 517 nm was measured after 30 min. The reducing power was determined by incubating 1 mL of sample with 1 mL of sodium phosphate buffer and potassium ferricyanide at 50 °C for 20 min. After centrifugation (6000× *g*, 10 min), the absorbance of the supernatant at 700 nm was recorded.

#### 2.2.7. Foaming Ability and Stability

The foaming ability and stability were measured by homogenizing 20 mL of a PPI or PPH solution (50 mg/mL) at 11,000 rpm for 2 min. The foaming ability was calculated as the volume of foam (Vf) divided by the initial volume (Vl). The foaming stability was calculated as the volume ratio after 30 min of quiescence.

### 2.3. Emulsion Preparation and Characterization

#### 2.3.1. Emulsion Preparation

Emulsions were prepared following the method described by Zhao et al. [43]. A total of 3 mg of the PPI, PPH, or sorbitan monostearate (SV) sample was dissolved in 27 mL of deionized water. Oil/water (O/W) emulsions were then prepared by homogenizing 9 mL of soybean oil with 27 mL of the PPI, PPH, or SV solution at a speed of 11,000 rpm.

#### 2.3.2. Emulsion Activity Index (EAI) and Emulsion Stability Index (ESI)

The EAI and ESI were estimated based on the turbidity of the samples following the method of Hu & Xiong [44]. At 0 and 15 min, 50 μL of freshly prepared emulsion was mixed with 5 mL of 0.1% SDS solution in a test tube and vortexed thoroughly. The absorbance of the diluted emulsion was measured at 500 nm. This was calculated based on the absorbance measurements at 500 nm.

$$\mathrm{EAI}\;(m^{2}/g)=\frac{2.303\;\times \;2\;\times \;A_{0}\;\times \;DF}{C\;\times \;\varphi \;\times \;L\;\times \;10,000}$$

$$\mathrm{ESI}\;(\min)=\frac{A_{0}∆t}{A_{0}\;-\;A_{15}}$$

A_0_: absorbance at 0 min; DF: dilution factor (1000); C: protein concentration in the aqueous phase (g/mL); φ: volume fraction of oil used in the emulsion (0.25); L: path length of the quartz cuvette (0.01 m); A_15_: absorbance at 15 min; and ∆t: 15 min.

#### 2.3.3. Particle Characteristics

The particle characteristics of the emulsions were evaluated following the method of Cheng et al. [45]. A 50 μL emulsion sample was diluted with 5 mL of 0.1 M phosphate-buffer solution to minimize the effects of multiple scattering. The diluted samples were analyzed at 25 °C using a nanoparticle size and zeta potential analyzer (Mastersizer Nano-2S, Malvern Panalytical, Malvern, UK) to determine the particle size, polydispersity index (PDI), and zeta potential. Particle size was expressed as the average diameter (z-average).

#### 2.3.4. Viscosity Measurement

The viscosity of the emulsion solution was measured with a rheometer (Physica MCR 92, Anton Paar, Ostildern, Germany). The apparent viscosity was recorded as a function of the shear rate (0.01–100 s^−1^) using the Rheoplus software (version 3.4, Anton Paar, Graz, Austria).

#### 2.3.5. Emulsion Morphology

The emulsion morphology was observed using an optical microscope (BH200, Sunny Optical Technology, Hangzhou, China) at 40× magnification.

### 2.4. Chiffon Rice Cake Preparation and Measurement

Chiffon rice cakes were prepared following the procedures described by Morisaki using the following ingredients: rice flour, whole milk, egg whites, egg yolk, soybean oil, sucrose, baking powder, lemon juice, and PPI/PPH/SV [46]. First, the 185 g of egg whites and 3 g of lemon juice were whipped for 1 min. A total of 110 g of sugar was then gradually added while continuing to whip for an additional 4 min. Once the meringue reached the soft peak stage, 100 g of egg yolks were incorporated and whipped for another 1 min. Subsequently, 100 g of rice flour was folded in using a spatula until no lumps remained. Baking powder (3 g); PPI, PPHs, or SV (2 g); milk (100 g); and soybean oil (38 g) were then sequentially added and mixed until the batter was homogeneous. The batter was poured into a round baking mold and baked at 170 °C for 30 min. After baking, the cake was inverted and allowed to cool completely before further processing.

The batter density was measured by dividing the weight of 100 mL of batter by its volume. The specific volume was calculated as described by Sangnark and Noomhorm [47]. The fully cooled cake was weighed to determine its mass (m), followed by measuring its volume (V) using the black sesame substitution method. The specific volume was calculated based on the volume and mass, where a higher value indicated a larger volume per unit of weight.

$$\mathrm{Specific}\;\mathrm{volume}\;(\mathrm{mL}/g)=\frac{V}{m}$$

According to the method of Paraskevopoulou et al., a texture profile analysis was conducted using a TA+^®^ Texture Analyzer (Stable micro systems, Goldalming, UK) to determine the hardness, cohesiveness, and resilience of the cake crumb [48]. The cake was cut into pieces of 2.5 cm × 2.5 cm × 2 cm in size, and a cylindrical probe with a 0.5-inch diameter (P/0.5) was used for texture profile analysis (TPA). The compression speed was 1 mm/s, with each compression reducing the sample height by 50%. The time delay between the first and second compression was 30 s.

### 2.5. Statistical Analysis

The data are presented as the mean ± standard deviation of the three replicates. The data were analyzed using a one-way analysis of variance (ANOVA) and Duncan’s multiple range test (*p* < 0.05) with SPSS software (version 30, SPSS, Armonk, NY, USA). The Principal Component Analysis (PCA) calculations were performed using PAST 4.03 (PAleontological STatistics). In addition, all data were standardized using Z-score normalization prior to PCA to eliminate the impact of differing value ranges among variables.

## 3. Results and Discussion

### 3.1. Hydrolysis Yields, Degrees of Hydrolysis, and Molecular Weights of PPI and PPHs

The hydrolysis yields of the PPHs are presented in Table 1. The yield was determined by comparing the lyophilized PPH mass to the original PPI weight. The hydrolysis yield increased with the enzyme treatment duration, with the highest yield observed after 4 h of papain hydrolysis (P4). There were no significant differences among the yields of the papain treatments from 0.25 to 2 h (P0.25, P0.5, P1, P2). Similarly, bromelain hydrolysis showed no significant yield variations from 0.25 to 2 h (B0.25, B0.5, B1, B2), although the yields were consistently lower than those of the papain-treated groups.

**Figure 1:**
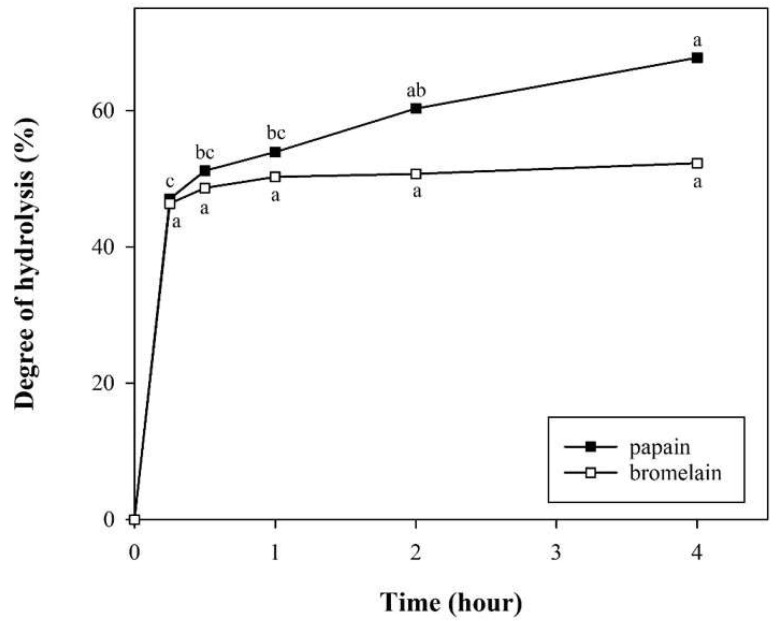
Degree of hydrolysis of potato protein hydrolysates (PPHs) obtained after 0.25, 0.5, 1, 2, and 4 h of hydrolysis with papain or bromelain. The values are the mean ± standard deviation of the three replicates. The different letters represent a significant (*p* < 0.05) difference between the different samples.

The degree of hydrolysis for both enzymes rapidly increased within the first 30 min (Figure 1), reaching a maximum after 4 h (67.76% for papain and 52.28% for bromelain). The rate of hydrolysis by bromelain slowed after 30 min, possibly due to a reduction in available peptide bonds, reaching a saturation point [49]. In contrast, papain maintained higher hydrolytic activity, resulting in more efficient protein breakdown.

The SDS-PAGE analysis revealed the molecular weight distributions of the PPI and PPHs. In the control (lane 0), PPI primarily consisted of patatin (42 kDa) and protease inhibitors (25 kDa), with some high-molecular-weight polypeptides (>100 kDa) likely corresponding to the starch phosphorylase L-1 [4]. Following hydrolysis, papain-treated PPH (Supplementary Figure S1A) showed the significant degradation of patatin after 15 min, with peptide fragments appearing at 15–20 kDa. After 30 min, the bands had almost entirely disappeared. Bromelain-treated PPH (Supplementary Figure S1B) showed similar trends, although the fragments remained larger (<11 kDa) until after 1 h of hydrolysis.

### 3.2. Protein Solubility, Antioxidant Capacity, Foaming Stability, and Foaminging Ability

**Figure 2:**
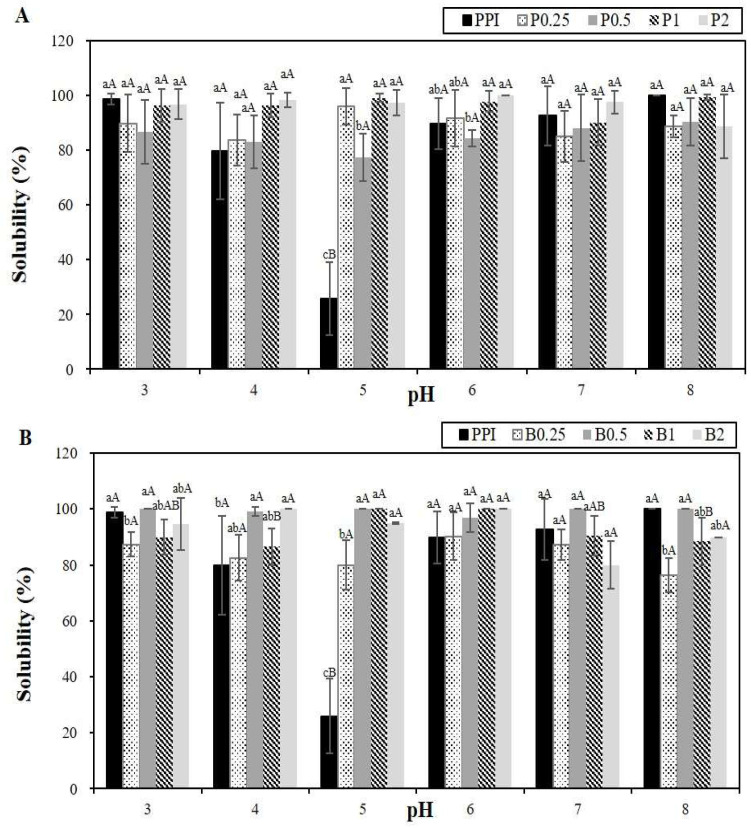
Effect of pH on the solubility (%) of potato protein isolate (PPI) and its hydrolysates (PPHs) obtained by hydrolysis with papain (**A**) and bromelain (**B**) for different lengths of time. The values are the mean ± standard deviation of the three replicates. ^a–c^ represent a significant (*p* < 0.05) difference between the different samples. ^A,B^ represent a significant (*p* < 0.05) difference between the different pH values. P0.25, PPH obtained after 15 min of hydrolysis with papain; P0.5, PPH obtained after 30 min of hydrolysis with papain; P1, PPH obtained after 1 h of hydrolysis with papain; P2, PPH obtained after 2 h of hydrolysis with papain; B0.25, PPH obtained after 15 min of hydrolysis with bromelain; B0.5, PPH obtained after 30 min of hydrolysis with bromelain; B1, PPH obtained after 1 h of hydrolysis with bromelain; and B2, PPH obtained after 2 h of hydrolysis with bromelain.

The protein solubility across pH 3 to pH 8 was evaluated (Figure 2). PPI showed low solubility in the pH range of 4–6, with the lowest solubility observed at pH 5 (25.89%), corresponding to its isoelectric point (pI) [50]. However, the solubilities of the PPHs from both papain and bromelain hydrolysis were higher than those of PPI, especially between pH 4 and pH 6. This improvement was attributed to the lower molecular weights of the hydrolysates, which enhanced their solubilities through increased hydrophilic interactions and the exposure of amino groups [51]. The increases in the solubility of PPHs suggested their potential applications in food and beverage formulations, particularly at pH 5.

**Figure 3:**
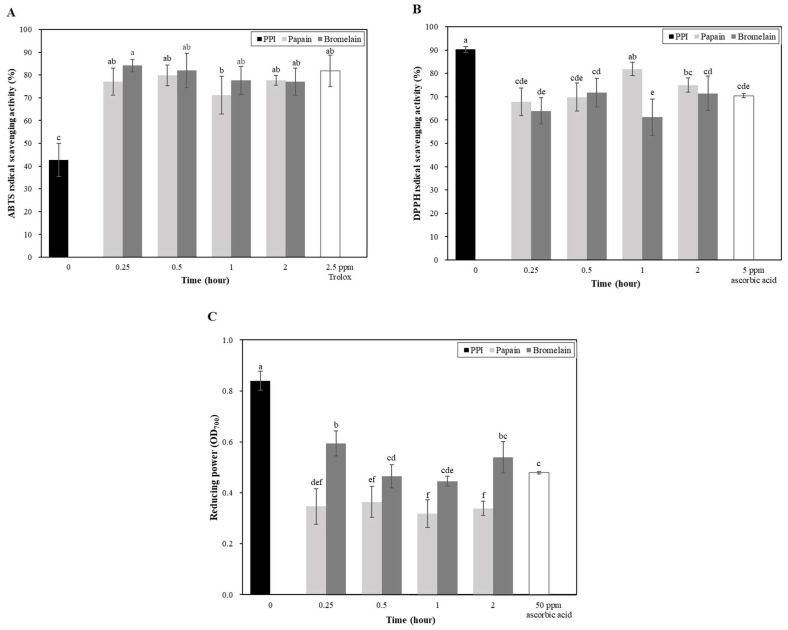
(**A**) ABTS radical-scavenging activity (%), (**B**) DPPH radical-scavenging activity (%), and (**C**) the reducing power of potato protein isolate (PPI) and potato protein hydrolysates (PPHs) obtained by hydrolysis with papain or bromelain for different lengths of time. The values are the mean ± standard deviation of the three replicates. The different letters represent a significant (*p* < 0.05) difference between the different samples. P0.25, PPH obtained after 15 min of hydrolysis with papain, P0.5, PPH obtained after 30 min of hydrolysis with papain; P1, PPH obtained after 1 h of hydrolysis with papain; P2, PPH obtained after 2 h of hydrolysis with papain; B0.25, PPH obtained after 15 min of hydrolysis with bromelain; B0.5, PPH obtained after 30 min of hydrolysis with bromelain; B1, PPH obtained after 1 h of hydrolysis with bromelain; and B2, PPH obtained after 2 h of hydrolysis with bromelain.

The antioxidant capacities of the PPI and PPHs were assessed using ABTS and DPPH assays. The ABTS radical-scavenging activity of PPI (2 mg/mL) was 42.69%, while the PPHs showed significantly higher activity, with no significant difference between the hydrolysates from the papain and bromelain treatments (Figure 3A). The PPHs demonstrated similar ABTS-scavenging abilities to 2.5 ppm Trolox, indicating their potential as natural antioxidants. Conversely, the DPPH-scavenging activity of PPI (90.28% at 10 mg/mL) was higher than that of the PPHs, which showed similar values to those of 5 ppm ascorbic acid (Figure 3B). Contrasting the results between the ABTS and DPPH assays likely reflected differences in solubility, with ABTS being water-soluble and DPPH being fat-soluble, as noted by Shahi et al. [52]. This suggests that hydrolysis by papain and bromelain may enhance the availability of hydrophilic antioxidant peptides for ABTS scavenging but reduce the hydrophobic peptides important for DPPH activity. Sbroggio et al. evaluated the antioxidant activity of protein hydrolysates obtained from okara using Alcalase and Flavourzyme [53]. The hydrolysates exhibited enhanced DPPH and ABTS radical-scavenging activity compared to the unhydrolyzed samples. For Alcalase hydrolysates, antioxidant activity increased to 99.5% for ABTS and 17.7% for DPPH, while Flavourzyme hydrolysates reached 88.2% for ABTS and 18.5% for DPPH [53]. In comparison, the PPHs in the present study exhibited even higher antioxidant activity, with the DPPH’s radical-scavenging capacities surpassing those reported for okara hydrolysates by Sbroggio et al. [53].

As shown in Figure 3C, at a protein concentration of 10 mg/mL, the absorbance value of PPI was 0.84, while that of PPH ranged between 0.32 and 0.60. Similar to the DPPH radical-scavenging ability, the reducing power of PPI was significantly higher than that of PPH and 50 ppm ascorbic acid, with no significant differences observed among the PPH results. Similar phenomena were also reported in studies on hemp protein hydrolyzed by Alcalase [28] and peanut protein [54], where the reducing power was highest at a low degree of hydrolysis. Furthermore, the study by Intarasirisawat et al. mentioned that both the degree of hydrolysis and protein concentration could potentially influence the results of antioxidant assays [55].

**Figure 4:**
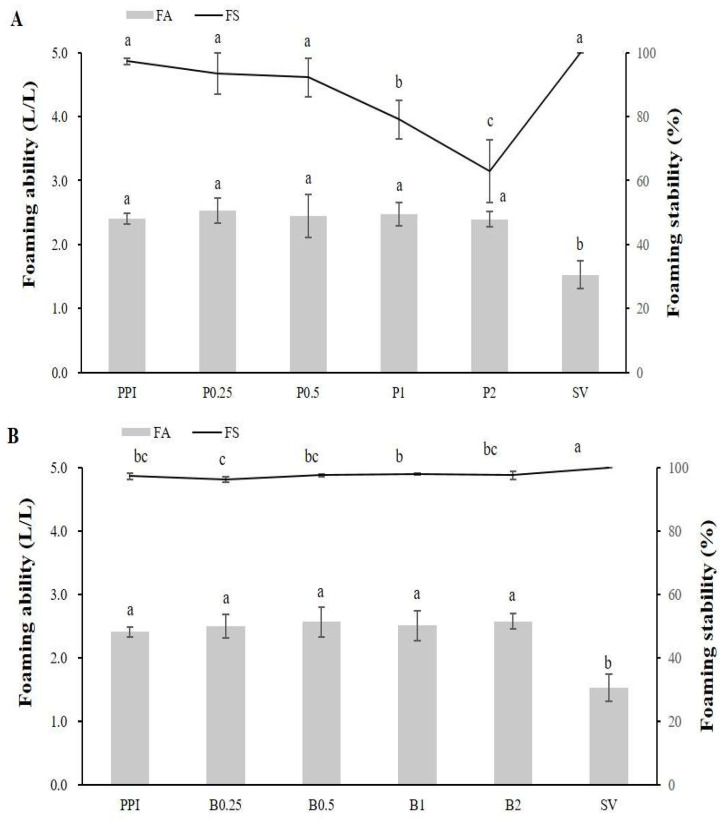
Foaming ability (L/L) and foaming stability (%) of potato protein isolate (PPI), sorbitan monostearate (SV), and potato protein hydrolysates (PPHs) obtained by hydrolysis with papain (**A**) and bromelain (**B**) for different lengths of time. The values are the mean ± standard deviation of the three replicates. The different letters represent a significant (*p* < 0.05) difference between the different samples. P0.25, PPH obtained after 15 min of hydrolysis with papain; P0.5, PPH obtained after 30 min of hydrolysis with papain; P1, PPH obtained after 1 h of hydrolysis with papain; P2, PPH obtained after 2 h of hydrolysis with papain; B0.25, PPH obtained after 15 min of hydrolysis with bromelain; B0.5, PPH obtained after 30 min of hydrolysis with bromelain; B1, PPH obtained after 1 h of hydrolysis with bromelain; B2, PPH obtained after 2 h of hydrolysis with bromelain; and SV, sorbitan monostearate made from vegetable (palm) fatty acids.

The foaming properties of the PPI and PPHs were compared (Figure 4). The foaming abilities of the PPI and PPHs ranged from 2.39 to 2.57 L/L, with no significant differences between treatments. The foaming stability of papain-treated PPHs decreased with longer hydrolysis times, particularly at P2, suggesting that over-hydrolysis reduced the foaming stability. In contrast, the foaming stability of bromelain-treated PPHs remained consistent. This observation aligns with previous reports indicating that extensive hydrolysis can increase protein solubility but reduce foaming stability due to changes in protein conformations [56].

### 3.3. Emulsion Activity, Stability, and Characteristics of the Emulsions

**Figure 5:**
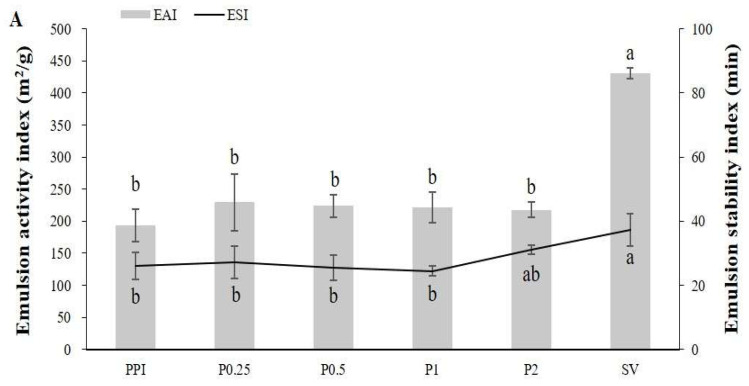

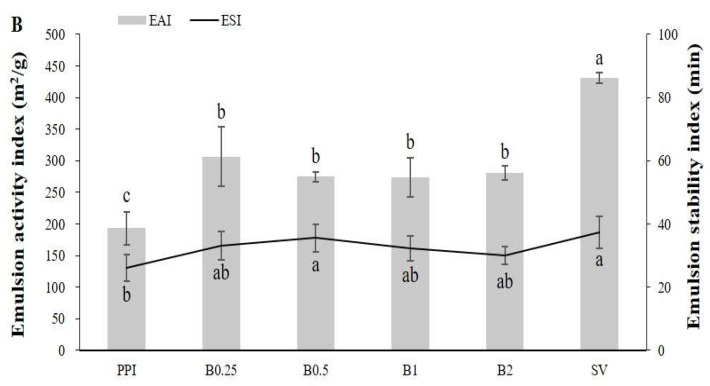
Emulsion activity index (m^2^/g) and emulsion stability index (min) of potato protein isolate (PPI), sorbitan monostearate (SV), and potato protein hydrolysates (PPHs) obtained by hydrolysis with papain (**A**) and bromelain (**B**) for different lengths of time. The values are the mean ± standard deviation of the three replicates. The different letters represent a significant (*p* < 0.05) difference between the different samples. P0.25, PPH obtained after 15 min of hydrolysis with papain; P0.5, PPH obtained after 30 min of hydrolysis with papain; P1, PPH obtained after 1 h of hydrolysis with papain; P2, PPH obtained after 2 h of hydrolysis with papain; B0.25, PPH obtained after 15 min of hydrolysis with bromelain; B0.5, PPH obtained after 30 min of hydrolysis with bromelain; B1, PPH obtained after 1 h of hydrolysis with bromelain; B2, PPH obtained after 2 h of hydrolysis with bromelain; and SV, sorbitan monostearate made from vegetable (palm) fatty acids.

EAI represents the ability of the proteins to assist in dispersing the oil phase into the water phase, while ESI indicates the capacity of proteins to maintain the emulsion in a dispersed state over time, preventing phenomena such as creaming, flocculation, or coalescence [57,58]. These emulsion parameters are presented in Figure 5. In Figure 5A, although the EAI shows an increase, there is no significant difference between the groups. As shown in Figure 5B, the PPHs obtained from bromelain hydrolysis exhibit the highest EAI value (306 m^2^/g) at 15 min (B0.25). The higher EAI observed in the bromelain-treated PPHs was attributed to the enhanced dispersibility of proteins at the O/W interface, while the decrease in ESI reflected the destabilization of the emulsions at higher hydrolysis levels. When hydrolyzed to a certain extent, the molecular weight of the peptide molecules decreased significantly, reducing their amphiphilic properties at the water–oil interface. This led to an increased accumulation of aggregated phases, thereby reducing stability [39]. SV exhibited the highest EAI and ESI, and the bromelain hydrolysis group exhibited higher EAI and ESI compared to the papain group, which may be attributed to partial protein hydrolysis exposing hidden hydrophobic groups, thereby improving the hydrophobic–hydrophilic balance and resulting in an enhanced emulsification performance [59].

As shown in Table 2, the order of the oil droplet sizes in the emulsions is as follows: papain-hydrolyzed PPH < PPI < bromelain-hydrolyzed PPH < SV. The Z-average of emulsions stabilized by bromelain-hydrolyzed proteins was larger than that of the PPI emulsions. The dynamic light scattering analysis revealed that PPHs from bromelain hydrolysis exhibited larger particle sizes (Z-average) than those of PPI, with a corresponding decrease in the PDI, indicating more heterogeneous emulsion particle sizes (Table 2). A higher PDI value in PPI was correlated with a reduced emulsion stability (Figure 5), with larger particle sizes tending to undergo flocculation and reduced uniformity [60]. Zeta potentials were more negative in PPHs compared to PPI, which enhanced the electrostatic repulsion between the particles and contributed to emulsion stability [61]. The microstructures of the emulsions prepared from the PPHs of papain and bromelain were observed under an optical microscope and are shown in Supplementary Figures S2 and S3. It can be seen that the emulsion droplets of all samples exhibited minimal aggregation, demonstrating good droplet dispensability. Consistent with the trends in the average droplet size changes shown in Table 2, the droplet sizes of PPH hydrolyzed by both enzymes began to increase after 1 h of hydrolysis.

The apparent viscosities of PPI, PPHs, and SV emulsions decreased with an increasing shear rate, exhibiting shear-thinning behavior (Table 3). The emulsions from SV and bromelain-treated PPHs showed higher viscosities compared to those of PPI and papain-treated PPHs. The viscosities of certain bromelain-treated PPHs remained significantly higher than the SV emulsions, which suggested stronger physical stability against coalescence and flocculation [62]. High viscosity greatly contributed to emulsion stability, as the viscous nature of emulsions could delay the gravitational separation and Brownian motion of the oil droplets. This reduced the frequency and efficiency of droplet collisions, thereby enhancing the physical stability of the emulsions and preventing creaming, flocculation, and coalescence [62].

### 3.4. Chiffon Rice Cake Batter Characteristics, Baking Performance, and Texture Analysis

Batter density is determined by measuring the weight of a known volume of batter and serves as a key indicator of air incorporation (aeration). Its value is related to the amount of air introduced during the batter mixing process [63]. The batter densities of rice chiffon cakes with different emulsifiers were measured (Table 4). The density of the PPH batters was lower than those of the PPI obtained from bromelain hydrolysis, correlating with the air incorporation in the batter. The specific volume ranging from 3.14 to 3.73 mL/g was higher for P1 and P2, as well as for B1 and B2. However, no significant difference was observed in the specific volume of the baked cakes, suggesting that air retention during baking was similar across samples. Specific volume represents the amount of air retained in the final baked product [63], with higher values indicating greater gas retention. The overall appearance of the cakes and the internal structure are shown in Figure 4 and Figure 5, respectively.

The textural properties of rice chiffon cakes with various emulsifiers were assessed (Table 5). Previous studies have shown that PPH typically reduces the hardness of food products [64]. However, the results of this study suggest that hydrolyzed proteins may instead contribute to increased cake hardness. The hardness of the cake with PPI was 0.49 ± 0.10 N, which increased significantly in the papain-hydrolyzed PPH cake to a range of 0.70–1.01 N. Similarly, the bromelain-hydrolyzed PPH cake exhibited increased hardness, ranging from 0.73 to 0.91 N, indicating that hydrolyzed proteins may have enhanced the cake hardness. Papain hydrolysis significantly increased the springiness from 0.815 to 0.861, while bromelain hydrolysis resulted in a significant increase to 0.841–0.855. SV demonstrated similar springiness compared to the PPH group. PPI exhibited the lowest cohesiveness at 0.763 ± 0.022. In contrast, PPH and SV showed cohesiveness in the range of 0.782–0.797, with no significant differences between the groups. PPHs demonstrated higher resilience compared to PPI (0.262 ± 0.008), with values ranging from 0.278 to 0.320. No significant differences were observed among the PPH group and SV. Additionally, cakes with PPHs hydrolyzed with papain and bromelain as the emulsifier had comparable hardness, springiness, cohesiveness, and resilience with SV, indicating that they were effective emulsifiers in gluten-free formulations. PCA was applied to evaluate the textural differences among PPI and PPH samples based on density, specific volume, hardness, springiness, cohesiveness, and resilience (Supplementary Figure S6). Variable loadings indicated that density and specific volume were the primary contributors to PC1, while cohesiveness and resilience contributed more to PC2, highlighting the multidimensional nature of textural variation across samples. Samples were separated based on formulation differences, with distinct clusters representing PPH samples with similar textural profiles to SV.

## 4. Conclusions

In this study, PPHs were prepared using papain and bromelain under varying hydrolysis times (0.25–2 h) to enhance their functional and emulsifying properties. Enzymatic hydrolysis effectively reduced the molecular weights of patatin (42 kDa) and the protease inhibitor (25 kDa), while significantly improving protein solubility under acidic conditions (pH 4–6). PPHs demonstrated higher ABTS radical-scavenging activity compared to untreated PPI. Notably, the highest emulsifying activity index (306 m^2^/g) was achieved with bromelain-hydrolyzed PPHs at 15 min in an oil-in-water emulsion system (1:3). Bromelain-treated PPHs also exhibited superior emulsifying properties, as indicated by their small PDIs, high zeta potential, and elevated viscosity values. Incorporating PPHs as surfactants in gluten-free chiffon rice cake batters reduced the batter densities and significantly enhanced the textural attributes, including springiness, cohesiveness, and resilience. Among the enzymatic treatments, bromelain-hydrolyzed PPHs demonstrated the greatest potential as plant-based emulsifiers, offering promising applications in food systems requiring improved emulsion stability and functionality. This study highlights the value of enzymatically modified potato proteins as sustainable and versatile alternatives to traditional emulsifiers in the development of innovative plant-based food products.

## Figures and Tables

**Table 1:** Hydrolysis yields of potato protein hydrolysates (PPHs).

	Yield (%)
P0.25	29.21 ± 4.96 ^bc^
P0.5	28.65 ± 3.31 ^c^
P1	31.68 ± 0.09 ^bc^
P2	34.88 ± 2.73 ^b^
P4	41.30 ± 1.47 ^a^
B0.25	11.58 ± 2.32 ^e^
B0.5	14.50 ± 3.03 ^e^
B1	15.36 ± 4.94 ^e^
B2	16.99 ± 0.14 ^de^
B4	21.64 ± 4.16 ^d^

Values are the mean ± standard deviation of the three replicates. Different letters represent a significant (*p* < 0.05) difference between different samples. P0.25, potato protein hydrolysate (PPH) obtained after 15 min of hydrolysis with papain; P0.5, PPH obtained after 30 min of hydrolysis with papain; P1, PPH obtained after 1 h of hydrolysis with papain; P2, PPH obtained after 2 h of hydrolysis with papain; P4, PPH obtained after 4 h of hydrolysis with papain; B0.25, PPH obtained after 15 min of hydrolysis with bromelain; B0.5, PPH obtained after 30 min of hydrolysis with bromelain; B1, PPH obtained after 1 h of hydrolysis with bromelain; B2, PPH obtained after 2 h of hydrolysis with bromelain; and B4, PPH obtained after 4 h of hydrolysis with bromelain.

**Table 2:** Characteristics of emulsion particles of potato protein isolate (PPI), potato protein hydrolysates (PPHs), and sorbitan monostearate (SV) obtained by hydrolysis with papain (A) and bromelain (B) for different lengths of time.

	Z-Average (nm)	Polydispersity Index	Zeta Potential (mV)
PPI	1205 ± 34.28 ^d^	0.701 ± 0.036 ^a^	−5.48 ± 0.48 ^f^
P0.25	954 ± 64.63 ^e^	0.612 ± 0.024 ^bc^	−8.71 ± 0.47 ^b^
P0.5	944 ± 97.42 ^e^	0.662 ± 0.008 ^ab^	−7.75 ± 0.25 ^bcd^
P1	1125 ± 36.25 ^d^	0.645 ± 0.032 ^ab^	−7.07 ± 0.32 ^de^
P2	1192 ± 30.92 ^d^	0.649 ± 0.023 ^ab^	−7.66 ± 0.65 ^bcde^
B0.25	1203 ± 46.87 ^d^	0.557 ± 0.002 ^c^	−8.28 ± 0.17 ^bc^
B0.5	1272 ± 97.17 ^cd^	0.614 ± 0.109 ^bc^	−7.31 ± 0.64 ^cde^
B1	1662 ± 86.49 ^b^	0.620 ± 0.049 ^abc^	−6.69 ± 0.38 ^e^
B2	1406 ± 158.0 ^c^	0.628 ± 0.018 ^abc^	−8.00 ± 0.41 ^bcd^
SV	1851 ± 50.15 ^a^	0.620 ± 0.037 ^abc^	−10.01 ± 0.60 ^a^

The values are the mean ± standard deviation of the three replicates. The different letters in a column represent a significant (*p* < 0.05) difference between the different samples. PPI, potato protein isolate; P0.25, potato protein hydrolysate (PPH) obtained after 15 min of hydrolysis with papain; P0.5, PPH obtained after 30 min of hydrolysis with papain; P1, PPH obtained after 1 h of hydrolysis with papain; P2, PPH obtained after 2 h of hydrolysis with papain; B0.25, PPH obtained after 15 min of hydrolysis with bromelain; B0.5, PPH obtained after 30 min of hydrolysis with bromelain; B1, PPH obtained after 1 h of hydrolysis with bromelain; B2, PPH obtained after 2 h of hydrolysis with bromelain; and SV, sorbitan monostearate made from vegetable (palm) fatty acids.

**Table 3:** Viscosity of emulsions of potato protein isolate (PPI), potato protein hydrolysates (PPHs), and sorbitan monostearate (SV) with different shear rates.

	1 s^−1^	10 s^−1^	20 s^−1^	40 s^−1^	60 s^−1^	80 s^−1^	100 s^−1^
PPI	152 ± 40.69 ^bc^	24.20 ± 6.52 ^ab^	14.85 ± 4.80 ^ab^	9.43 ± 2.14 ^ab^	6.63 ± 1.77 ^ab^	5.09 ± 1.11 ^ab^	4.26 ± 0.85 ^a^
P0.25	0.18 ± 0.03 ^d^	0.16 ± 0.02 ^c^	0.13 ± 0.02 ^c^	0.11 ± 0.04 ^c^	0.09 ± 0.05 ^c^	0.09 ± 0.03 ^d^	0.08 ± 0.03 ^d^
P0.5	0.12 ± 0.09 ^d^	0.10 ± 0.05 ^c^	0.09 ± 0.04 ^c^	0.08 ± 0.03 ^c^	0.06 ± 0.03 ^c^	0.06 ± 0.02 ^d^	0.06 ± 0.02 ^d^
P1	0.20 ± 0.02 ^d^	0.17 ± 0.01 ^c^	0.14 ± 0.00 ^c^	0.13 ± 0.01 ^c^	0.11 ± 0.01 ^c^	0.10 ± 0.01 ^d^	0.10 ± 0.01 ^d^
P2	0.21 ± 0.03 ^d^	0.18 ± 0.02 ^c^	0.17 ± 0.02 ^c^	0.14 ± 0.01 ^c^	0.12 ± 0.01 ^c^	0.11 ± 0.00 ^d^	0.10 ± 0.00 ^d^
B0.25	97.82 ± 56.08 ^cd^	14.24 ± 7.12 ^bc^	7.54 ± 4.02 ^bc^	3.91 ± 1.51 ^c^	2.70 ± 1.36 ^c^	2.22 ± 0.86 ^cd^	1.89 ± 0.81 ^cd^
B0.5	98.06 ± 58.50 ^cd^	13.67 ± 5.76 ^bc^	7.49 ± 3.75 ^bc^	5.04 ± 1.34 ^bc^	3.46 ± 1.70 ^bc^	2.76 ± 0.53 ^bc^	2.22 ± 0.40 ^bc^
B1	277 ± 93.00 ^a^	37.42 ± 11.00 ^a^	20.39 ± 5.71 ^a^	10.44 ± 3.64 ^a^	7.38 ± 2.46 ^a^	5.47 ± 1.73 ^a^	4.61 ± 1.43 ^a^
B2	248 ± 48.48 ^ab^	32.02 ± 5.70 ^a^	17.87 ± 3.20 ^a^	9.09 ± 1.25 ^ab^	6.54 ± 1.33 ^ab^	4.81 ± 0.73 ^ab^	3.97 ± 0.67 ^ab^
SV	204 ± 97.82 ^abc^	33.54 ± 10.50 ^a^	19.45 ± 5.76 ^a^	9.93 ± 2.65 ^ab^	7.41 ± 2.37 ^a^	5.97 ± 1.47 ^a^	5.23 ± 1.14 ^a^

The values are the mean ± standard deviation of the three replicates. The different letters in a column represent a significant (*p* < 0.05) difference between the different samples. PPI, potato protein isolate; P0.25, potato protein hydrolysate (PPH) obtained after 15 min of hydrolysis withy papain; P0.5, PPH obtained after 30 min of hydrolysis with papain; P1, PPH obtained after 1 h of hydrolysis with papain; P2, PPH obtained after 2 h of hydrolysis with papain; B0.25, PPH obtained after 15 min of hydrolysis with bromelain; B0.5, PPH obtained after 30 min of hydrolysis with bromelain; B1, PPH obtained after 1 h of hydrolysis with bromelain; B2, PPH obtained after 2 h of hydrolysis with bromelain; and SV, sorbitan monostearate made from vegetable (palm) fatty acids.

**Table 4:** Batter density and specific volume of gluten-free rice cakes.

	Batter Density (g/cm^3^)	Specific Volume (mL/g)
PPI	0.451 ± 0.017 ^a^	3.21 ± 0.42 ^a^
P0.25	0.429 ± 0.012 ^ab^	3.36 ± 0.25 ^a^
P0.5	0.423 ± 0.015 ^ab^	3.25 ± 0.11 ^a^
P1	0.422 ± 0.020 ^ab^	3.73 ± 0.46 ^a^
P2	0.419 ± 0.012 ^b^	3.38 ± 0.59 ^a^
B0.25	0.398 ± 0.011 ^b^	3.14 ± 0.10 ^a^
B0.5	0.405 ± 0.020 ^b^	3.16 ± 0.18 ^a^
B1	0.421 ± 0.013 ^b^	3.71 ± 0.48 ^a^
B2	0.420 ± 0.020 ^b^	3.62 ± 0.13 ^a^
SV	0.422 ± 0.011 ^ab^	3.37 ± 0.24 ^a^

The values are the mean ± standard deviation of the three replicates. The different letters in a column represent a significant (*p* < 0.05) difference between the different samples. PPI, potato protein isolate; P0.25, potato protein hydrolysate (PPH) obtained after 15 min of hydrolysis with papain; P0.5, PPH obtained after 30 min of hydrolysis with papain; P1, PPH obtained after 1 h of hydrolysis with papain; P2, PPH obtained after 2 h of hydrolysis with papain; B0.25, PPH obtained after 15 min of hydrolysis with bromelain; B0.5, PPH obtained after 30 min of hydrolysis with bromelain; B1, PPH obtained after 1 h of hydrolysis with bromelain; B2, PPH obtained after 2 h of hydrolysis with bromelain; and SV, sorbitan monostearate made from vegetable (palm) fatty acids.

**Table 5:** Textural parameters of gluten-free rice cakes.

	Hardness (N)	Springiness	Cohesiveness	Resilience
PPI	0.49 ± 0.10 ^c^	0.777 ± 0.011 ^c^	0.763 ± 0.022 ^b^	0.262 ± 0.008 ^c^
P0.25	0.70 ± 0.05 ^bc^	0.815 ± 0.026 ^b^	0.779 ± 0.012 ^ab^	0.278 ± 0.016 ^bc^
P0.5	1.01 ± 0.30 ^a^	0.861 ± 0.002 ^a^	0.786 ± 0.002 ^ab^	0.317 ± 0.010 ^a^
P1	0.77 ± 0.06 ^abc^	0.849 ± 0.013 ^a^	0.793 ± 0.019 ^a^	0.307 ± 0.006 ^a^
P2	0.89 ± 0.20 ^ab^	0.853 ± 0.013 ^a^	0.797 ± 0.012 ^a^	0.304 ± 0.004 ^a^
B0.25	0.91 ± 0.10 ^ab^	0.848 ± 0.022 ^a^	0.782 ± 0.007 ^ab^	0.301 ± 0.016 ^ab^
B0.5	0.83 ± 0.08 ^ab^	0.855 ± 0.007 ^a^	0.784 ± 0.002 ^ab^	0.306 ± 0.010 ^a^
B1	0.73 ± 0.21 ^abc^	0.841 ± 0.032 ^ab^	0.789 ± 0.005 ^ab^	0.302 ± 0.027 ^ab^
B2	0.81 ± 0.13 ^ab^	0.850 ± 0.003 ^a^	0.787 ± 0.008 ^ab^	0.314 ± 0.011 ^a^
SV	0.89 ± 0.11 ^ab^	0.866 ± 0.016 ^a^	0.792 ± 0.008 ^a^	0.320 ± 0.010 ^a^

The values are the mean ± standard deviation of the three replicates. The different letters in a column represent a significant (*p* < 0.05) difference between the different samples. PPI, potato protein isolate; P0.25, potato protein hydrolysate (PPH) obtained after 15 min of hydrolysis with papain; P0.5, PPH obtained after 30 min of hydrolysis with papain; P1, PPH obtained after 1 h of hydrolysis with papain; P2, PPH obtained after 2 h of hydrolysis with papain; B0.25, PPH obtained after 15 min of hydrolysis with bromelain; B0.5, PPH obtained after 30 min of hydrolysis with bromelain; B1, PPH obtained after 1 h of hydrolysis with bromelain; B2, PPH obtained after 2 h of hydrolysis with bromelain; and SV, sorbitan monostearate made from vegetable (palm) fatty acids.

## Data Availability

The original contributions presented in the study are included in the article, further inquiries can be directed to the corresponding author.

## Supplementary Materials

The following supporting information can be downloaded at: https://www.mdpi.com/article/10.3390/foods14060978/s1, Figure S1: SDS-PAGE analysis of potato protein isolate (PPI) and its hydrolysates (PPHs) obtained by hydrolysis with papain (A) and bromelain (B) for different times. MW, molecular weight markers of 11–245 kDa; Lane, 0–2 h of enzymatic hydrolysis; Figure S2: Optical micrograph images of emulsions of potato protein isolate (PPI), sorbitan monostearate (SV), and potato protein hydrolysates (PPHs) obtained by hydrolysis with papain for different times (40×). Red scale lines are 20 μm. (A) PPI; (B) P0.25, PPH obtained after 15 min of hydrolysis with papain; (C) P0.5, PPH obtained after 30 min of hydrolysis with papain; (D) P1, PPH obtained after 1 h of hydrolysis with papain; (E) P2, PPH obtained after 2 h of hydrolysis with papain; (F) SV, sorbitan monostearate made from vegetable (palm) fatty acids; Figure S3: Optical micrograph images of emulsions of potato protein isolate (PPI), sorbitan monostearate (SV), and potato protein hydrolysates (PPHs) obtained by hydrolysis with bromelain for different times (40×). Red scale lines are 20 μm. (A) PPI; (B) B0.25, PPH obtained after 15 min of hydrolysis with bromelain; (C) B0.5, PPH obtained after 30 min of hydrolysis with bromelain; (D) B1, PPH obtained after 1 h of hydrolysis with bromelain; (E) B2, PPH obtained after 2 h of hydrolysis with bromelain; (F) SV, sorbitan monostearate made from vegetable (palm) fatty acids; Figure S4: Appearance of gluten-free rice cake. (A) PPI: potato protein isolate, (B) P0.25: PPH obtained by papain after 15 min of hydrolysis, (C) P0.5: PPH obtained by papain after 30 min of hydrolysis, (D) P1: PPH obtained by papain after 1 h of hydrolysis, (E) P2: PPH obtained by papain after 2 h of hydrolysis, (F) B0.25: PPH obtained by bromelain after 15 min of hydrolysis, (G) B0.5: PPH obtained by bromelain after 30 min of hydrolysis, (H) B1: PPH obtained by bromelain after 1 h of hydrolysis, (I) B2: PPH obtained by bromelain after 2 h of hydrolysis, (J) SV: sorbitan monostearate; Figure S5: Crumb structure of gluten-free rice cake. (A) PPI: potato protein isolate, (B) P0.25: PPH obtained by papain after 15 min of hydrolysis, (C) P0.5: PPH obtained by papain after 30 min of hydrolysis, (D) P1: PPH obtained by papain after 1 h of hydrolysis, (E) P2: PPH obtained by papain after 2 h of hydrolysis, (F) B0.25: PPH obtained by bromelain after 15 min of hydrolysis, (G) B0.5: PPH obtained by bromelain after 30 min of hydrolysis, (H) B1: PPH obtained by bromelain after 1 h of hydrolysis, (I) B2: PPH obtained by bromelain after 2 h of hydrolysis, (J) SV: sorbitan monostearate; Figure S6: Principal Component Analysis (PCA) plot of gluten-free rice cake made with potato protein isolate (PPI), potato protein hydrolysates (PPHs), or SV (sorbitan monostearate).
