# Supplementary material for: Enhancing the Functional and Emulsifying Properties of Potato Protein via Enzymatic Hydrolysis with Papain and Bromelain for Gluten-Free Cake Emulsifiers
Source: Foods. 2025 Mar 13;14(6):978. doi: 10.3390/foods14060978 (PMC11941777; doi:10.3390/foods14060978)
Supplement: Supplementary file 1 [file foods-14-00978-s001.zip › foods-3512833-supplementary.pdf]

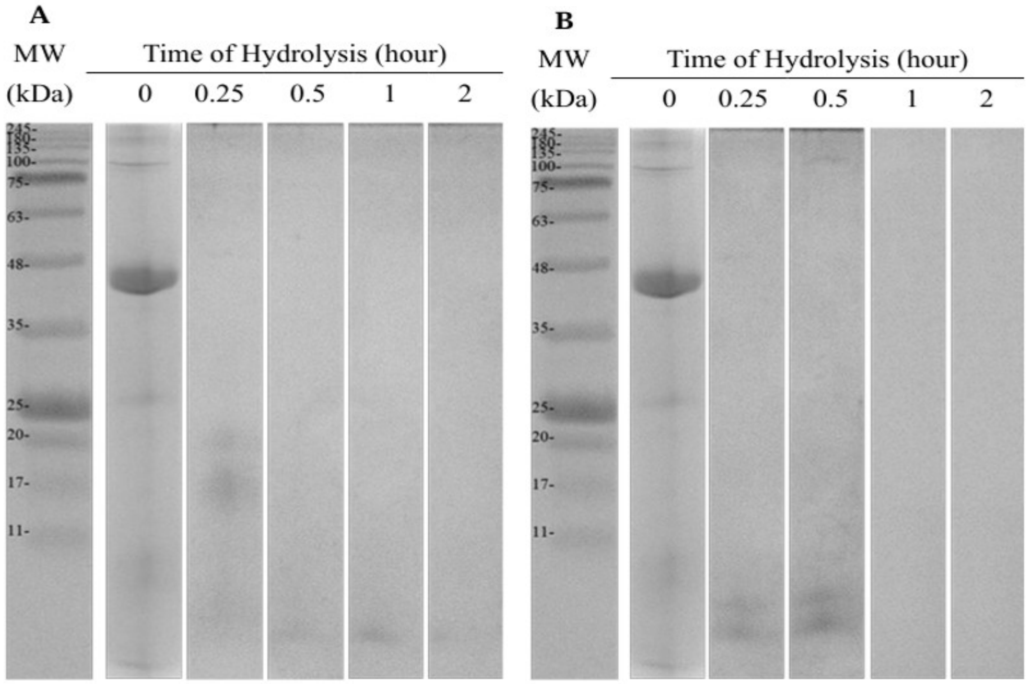

Supplementary Figure S1. SDS-PAGE analysis of potato protein isolate (PPI) and its hydrolysates (PPHs) obtained by hydrolysis with papain (A) and bromelain (B) for different times. MW, molecular weight markers of 11–245 kDa; Lane, 0–2 h of enzymatic hydrolysis.

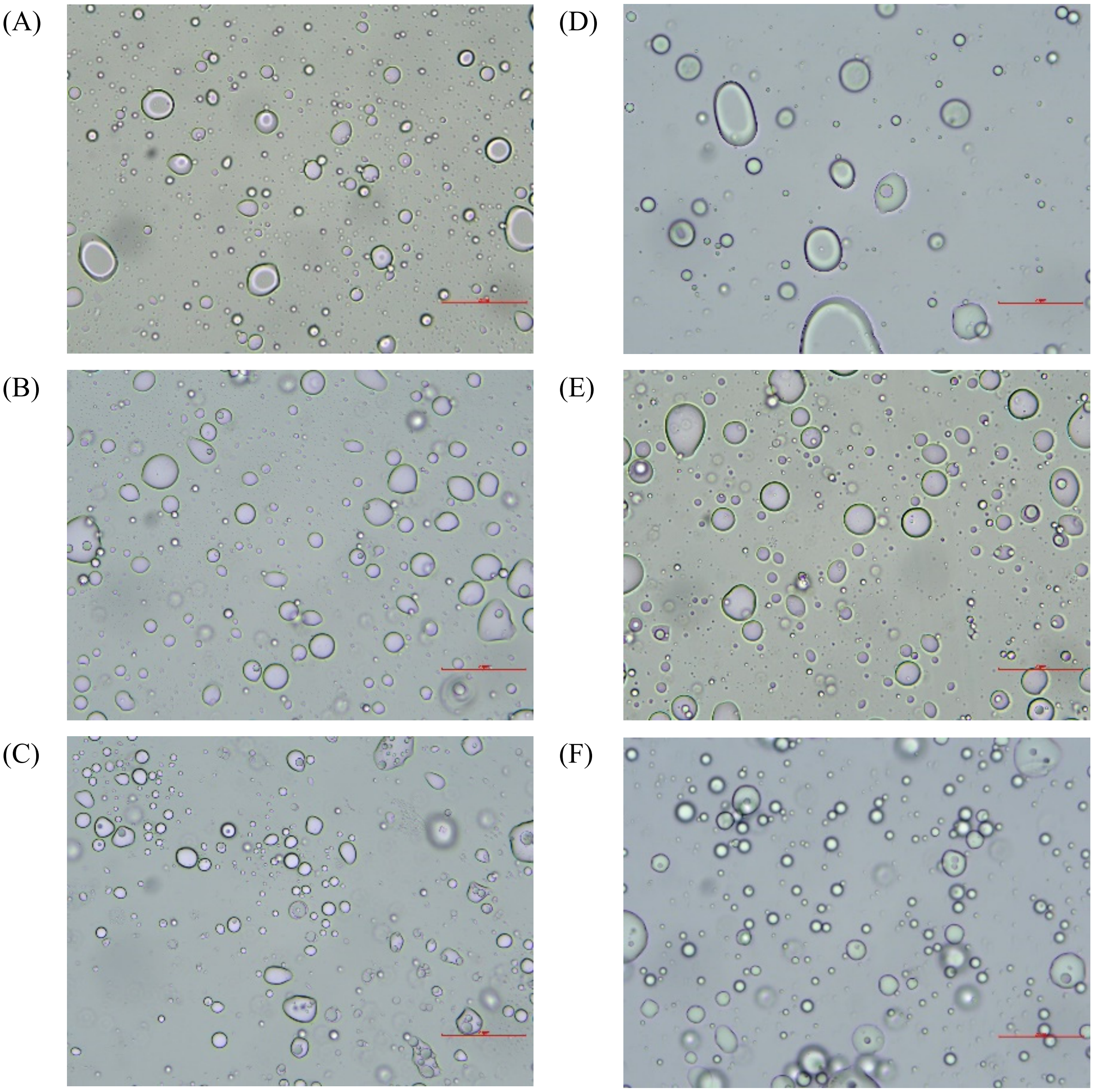

Supplementary Figure S2. Optical micrograph images of emulsions of potato protein isolate (PPI), sorbitan monostearate (SV), and potato protein hydrolysates (PPHs) obtained by hydrolysis with papain for different times (40×). Red scale lines are 20 μm. (A) PPI; (B) P0.25, PPH obtained after 15 min of hydrolysis with papain; (C) P0.5, PPH obtained after 30 min of hydrolysis with papain; (D) P1, PPH obtained after 1 h of hydrolysis with papain; (E) P2, PPH obtained after 2 h of hydrolysis with papain; (F) SV, sorbitan monostearate made from vegetable (palm) fatty acids.

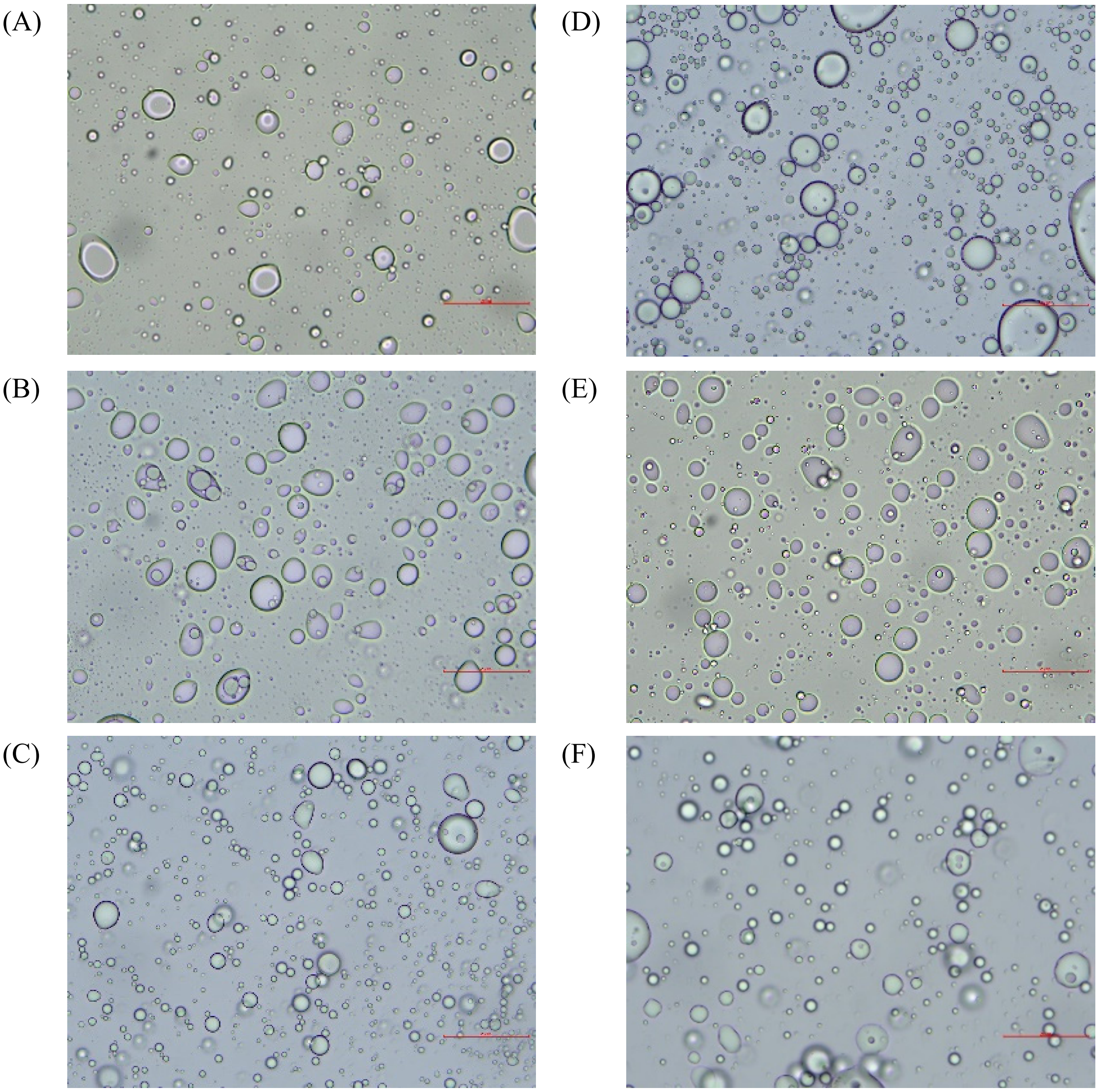

Supplementary Figure S3. Optical micrograph images of emulsions of potato protein isolate (PPI), sorbitan monostearate (SV), and potato protein hydrolysates (PPHs) obtained by hydrolysis with bromelain for different times (40×). Red scale lines are 20 µm. (A) PPI; (B) B0.25, PPH obtained after 15 min of hydrolysis with bromelain; (C) B0.5, PPH obtained after 30 min of hydrolysis with bromelain; (D) B1, PPH obtained after 1 h of hydrolysis with bromelain; (E) B2, PPH obtained after 2 h of hydrolysis with bromelain; (F) SV, sorbitan monostearate made from vegetable (palm) fatty acids.

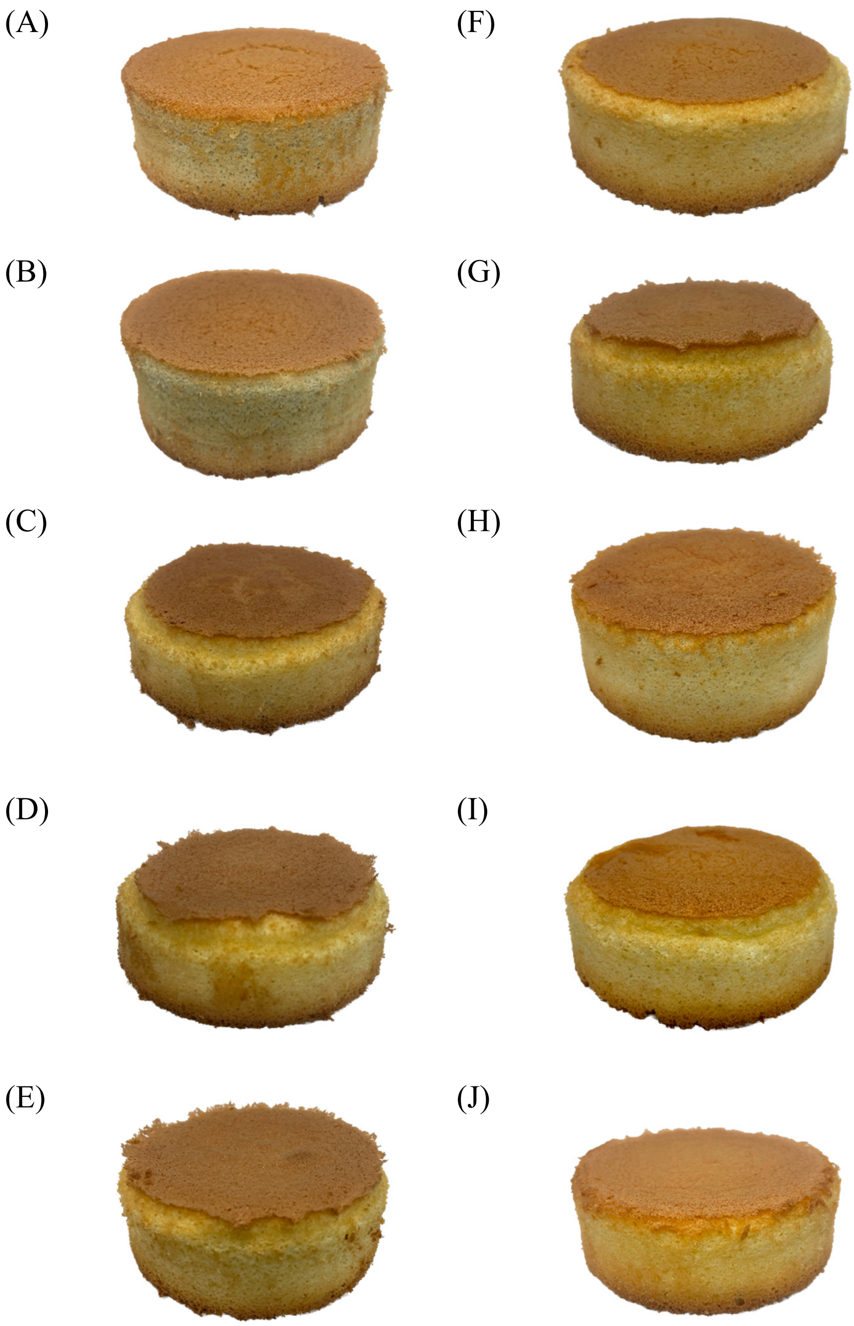

Supplementary Figure S4. Appearance of gluten-free rice cake. (A) PPI: potato protein isolate, (B) P0.25: PPH obtained by papain after 15 min of hydrolysis, (C) P0.5: PPH obtained by papain after 30 min of hydrolysis, (D) P1: PPH obtained by papain after 1 h of hydrolysis, (E) P2: PPH obtained by papain after 2 h of hydrolysis, (F) B0.25: PPH obtained by bromelain after 15 min of hydrolysis, (G) B0.5: PPH obtained by bromelain after 30 min of hydrolysis, (H) B1: PPH obtained by bromelain after 1 h of hydrolysis, (I) B2: PPH obtained by bromelain after 2 h of hydrolysis, (J) SV: sorbitan monostearate.

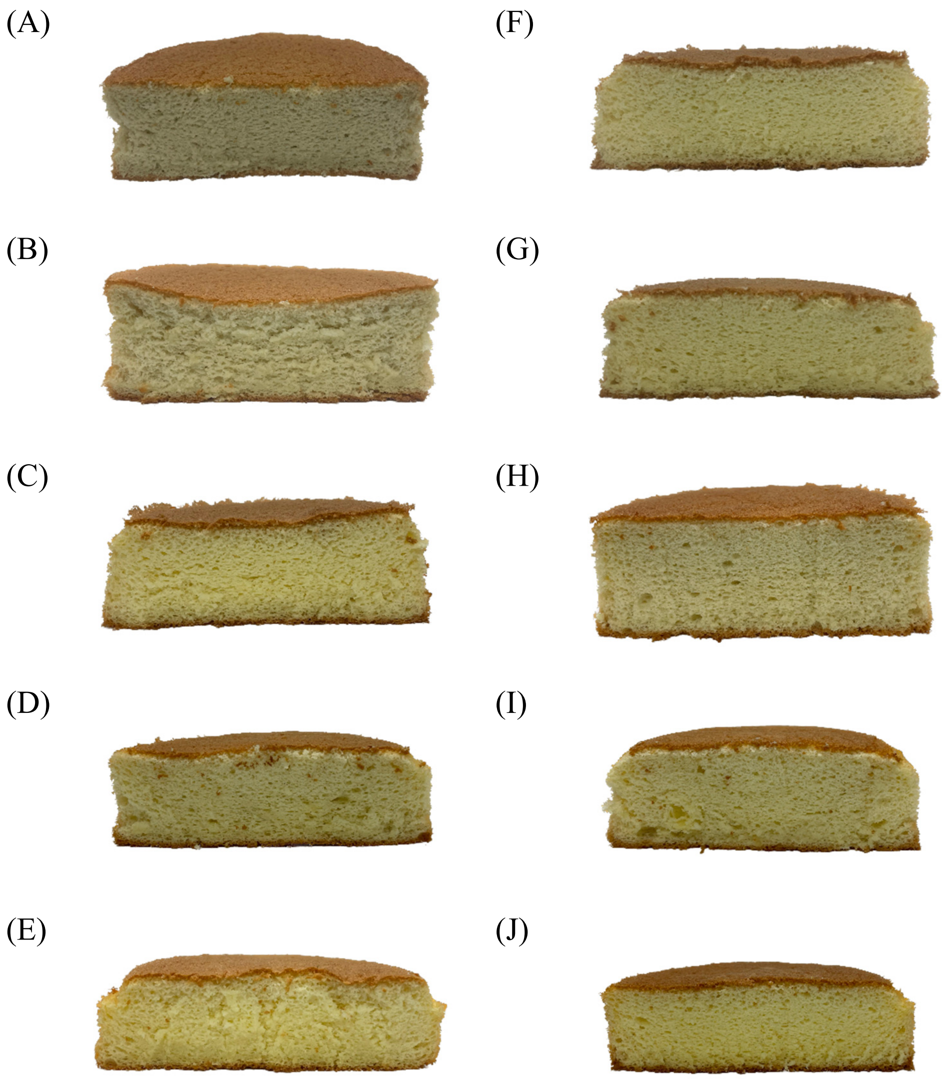

Supplementary Figure S5. Crumb structure of gluten-free rice cake. (A) PPI: potato protein isolate, (B) P0.25: PPH obtained by papain after 15 min of hydrolysis, (C) P0.5: PPH obtained by papain after 30 min of hydrolysis, (D) P1: PPH obtained by papain after 1 h of hydrolysis, (E) P2: PPH obtained by papain after 2 h of hydrolysis, (F) B0.25: PPH obtained by bromelain after 15 min of hydrolysis, (G) B0.5: PPH obtained by bromelain after 30 min of hydrolysis, (H) B1: PPH obtained by bromelain after 1 h of hydrolysis, (I) B2: PPH obtained by bromelain after 2 h of hydrolysis, (J) SV: sorbitan monostearate.

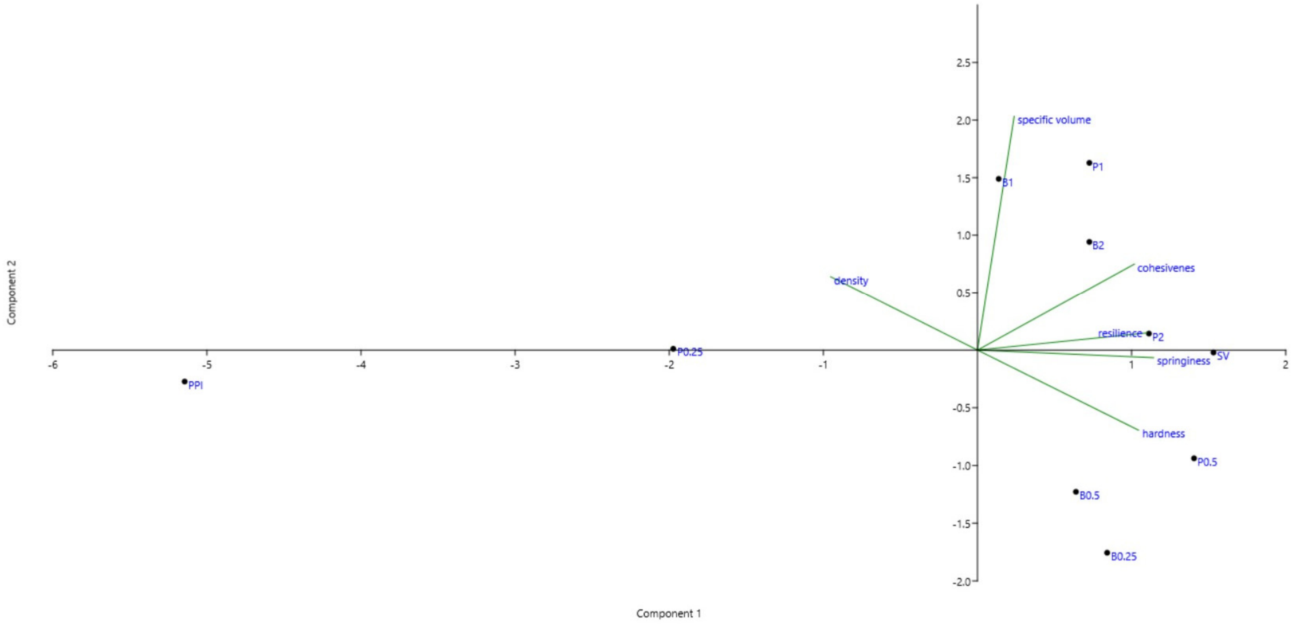

Supplementary Figure S6. Principal Component Analysis (PCA) plot of gluten-free rice cake made with potato protein isolate (PPI), potato protein hydrolysates (PPHs), or SV (sorbitan monostearate)
